# Supplementary material for: Identification of photoperiod-induced specific miRNAs in the adrenal glands of Sunite sheep (Ovis aries)
Source: Front Vet Sci. 2022 Jul 22;9:888207. doi: 10.3389/fvets.2022.888207 (PMC9354845; doi:10.3389/fvets.2022.888207)
Supplement: Supplementary file 1 [file Data_Sheet_1.ZIP › Supplementary file/miRDeep2 file.zip!/miRDeep2 file/Know_Structure/LP42A3/oar-mir-103.pdf]

miRBase precursor	: oar-mir-103
Total read count	: 21983
oar-miR-103 read count	: 21983
remaining reads	: 0

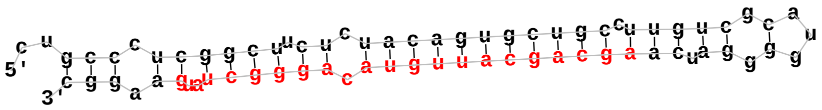

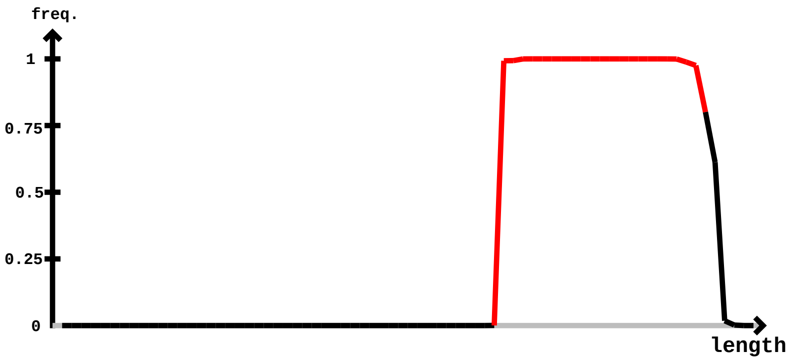


```
                                              oar-miR-103
5'- cugcccucggcuucucuacagugcugccuugucgcauggggaucaagcagcauuguacagggcuaugaaggc   -3'    exp
    ..(((.((((((.((.(((((((((((.(((((.(...).)).)))))))))))))).))))))..)).)))    reads      mm         sample
    ............................................aagcagcauuguacagggcuaug.....    5          0          seq
    ............................................aagcagcauuguacagggcuauga....    7          0          seq
    .............................................agcagcauuguacaggg..........    3          0          seq
    .............................................agcagcauuguacagggc.........    10         0          seq
    .............................................agcagcauuguacagggcu........    248        0          seq
    .............................................agcagcauuguacagggcua.......    281        0          seq
    .............................................agcagcauuguacagggcuau......    3838       0          seq
    .............................................agcagcauuguacagggcuaug.....    4108       0          seq
    .............................................agcagcauuguacagggcuauga....    12946      0          seq
    .............................................agcagcauuguacagggcuaugaa...    342        0          seq
    .............................................agcagcauuguacagggcuaugaag..    3          0          seq
    .............................................agcagcauuguacagggcuaugaaA..    36         1          seq
    ..............................................gcagcauuguacagggcuau......    2          0          seq
    ..............................................gcagcauuguacagggcuaug.....    1          0          seq
    ..............................................gcagcauuguacagggcuauga....    10         0          seq
    ..............................................gcagcauuguacagggcuaugaa...    1          0          seq
    ...............................................cagcauuguacagggcuau......    2          0          seq
    ...............................................cagcauuguacagggcuaug.....    18         0          seq
    ...............................................cagcauuguacagggcuauga....    104        0          seq
    ...............................................cagcauuguacagggcuaugaa...    9          0          seq
    ...............................................cagcauuguacagggcuaugaaA..    3          1          seq
    ...............................................cagcauuguacagggcuaugaag..    2          0          seq
    ................................................agcauuguacagggcuauga....    4          0          seq
```
